# Cleaning validation in pharmaceutical quality control laboratories: a structured protocol for contamination risk mitigation

**DOI:** 10.1007/s40199-025-00566-x

**Published:** 2025-07-03

**Authors:** Maria J. Moura, André D. Pereira, Daniel J. F. Santos, Ana G. Silva, Carla C. A. D. Paiva, Belmiro P. M. Duarte

**Affiliations:** 1 Departamento de Engenharia Química e Biológica, Instituto Superior de Engenharia – Instituto Politécnico de Coimbra, Rua Pedro Nunes, Coimbra, 3030-199 Portugal; 2 CERES, Universidade de Coimbra, Rua Sílvio Lima — Pólo II, Coimbra, 3030–790 Portugal; 3 Medinfar Manufacturing, S.A., Pq. Ind. Amando Martins Tavares, Rua do Outeiro da Armada n. 5, Condeixa-a-Nova, 3150–194 – Sebal Portugal; 4 INESC Coimbra, Universidade de Coimbra, Rua Sílvio Lima — Pólo II, Coimbra, 3030–790 Portugal

**Keywords:** Cleaning validation protocol, Laboratory equipment, Systematic approach, Recovery study

## Abstract

**Background:**

Cleaning activities are critical in pharmaceutical manufacturing to prevent cross-contamination of Active Pharmaceutical Ingredients (APIs). Traditionally, cleaning validation protocols have focused on production lines. However, there is a growing trend toward extending these protocols to Quality Control (QC) laboratories, encompassing both glassware and stainless-steel equipment.

**Objectives:**

This paper presents a systematic approach for developing cleaning validation protocols specifically designed for QC laboratory equipment, aimed at improving cleaning effectiveness and ensuring regulatory compliance.

**Methods:**

The proposed methodology includes: (i) identifying the worst-case API; (ii) performing recovery studies to optimize sampling methods and solvent selection; and (iii) employing statistical tools such as descriptive analysis and hypothesis testing to refine the protocol in line with current industry standards.

**Results:**

A case study involving Oxcarbazepine demonstrates the application of the proposed protocol, evaluating surface contamination across various QC instruments and assessing detergent residues to validate cleaning effectiveness.

**Conclusion:**

The proposed strategy provides a structured, statistically grounded framework for developing cleaning validation protocols in QC laboratories, promoting effective contamination control and adherence to regulatory standards.

**Graphical abstract:**

**Figure:**
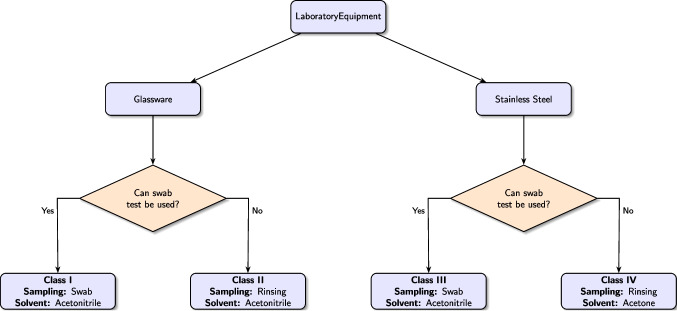
Decision tree for selecting the appropriate cleaning procedure for each equipment type

## Motivation

The importance of cleanliness in the pharmaceutical industry is paramount. Stricter cleaning requirements imposed by regulatory bodies have driven companies to adopt more practical, efficient, and optimized procedures. These measures aim to prevent contamination and cross-contamination before, during, and after manufacturing operations, based on the understanding that effective cleaning significantly reduces contamination risks.

Over the last two decades, cleaning practices have evolved substantially and are now considered on par with validated manufacturing processes [1]. This shift is driven by: (i) the emergence of highly potent drugs, (ii) recent contamination incidents, and (iii) the rise of personalized medicine, which acknowledges varying patient sensitivities [2].

Cleaning validation ensures the removal of active substances, excipients, cleaning agents, and microbial contaminants to acceptable levels [3]. It is guided by regulatory frameworks [4–7] and supported by established scientific literature [8–12]. However, a nuanced scientific approach remains essential, especially as validation practices extend to quality control (QC) laboratories in both production and development settings [13–16].

Extending cleaning validation protocols to laboratory equipment presents unresolved challenges, and the current literature lacks a comprehensive, systematic approach to address them. To our knowledge, key aspects such as:

A. selecting the Active Pharmaceutical Ingredient (API) to anchor the study;
B. choosing an appropriate solvent;
C. identifying suitable sampling methods for diverse lab equipment; and
D. selecting analytical techniques for residue detection;

remain insufficiently explored. Any effective strategy must reflect real-world industrial practices and incorporate the complexity of actual decision-making. This paper addresses this gap by proposing: (i) a systematic framework tackling aspects A–D; and (ii) a decision-making approach grounded in current industrial workflows.

Our contribution introduces three novel elements: (i) a structured protocol for developing cleaning validation procedures; (ii) a recovery study supporting solvent and sampling method selection; and (iii) a real-world case study applying the method to various QC lab equipment, including both glassware and stainless steel.

## Materials and methods

**Fig. 1:**
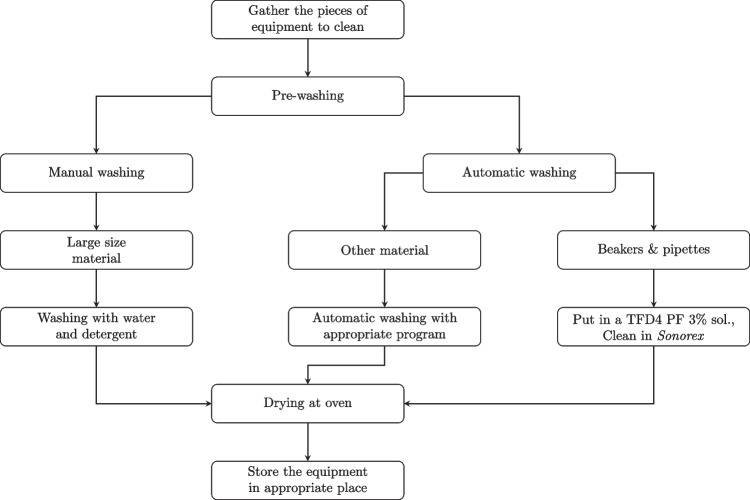
Current laboratory cleaning protocol for glassware and stainless-steel equipment

To broaden the study’s applicability, both glassware and stainless-steel laboratory equipment were considered. The current cleaning process distinguishes between manually and automatically cleaned items. Manually cleaned items are washed by hand using a phosphate-free alkaline detergent (TFD4 PF, *Franklab*), while automated cleaning uses an industrial washer with a standard program and TFD7 PF detergent (*Franklab*). After washing, all materials are dried in an oven at 60 $^{\circ }$C. The complete procedure is illustrated in Fig. 1 and applies to both equipment types.

### API selection

A worst-case scenario approach was adopted to select the API, consistent with established practices [17–19]. Selection criteria, defined in collaboration with the partner pharmaceutical company, include: (i) API concentration; (ii) solubility in water; (iii) solubility in acids and/or bases; (iv) toxicity; (v) cleaning difficulty; and (vi) cleaning method (manual or automatic) [20]. Low water solubility is directly associated with greater cleaning difficulty. Based on these criteria, Oxcarbazepine—an anticonvulsant with a history of cleaning challenges—was identified as the worst-case API. Its prior association with persistent contamination at the partnering company reinforces this choice.

The rationale is that a cleaning protocol effective against the most difficult-to-remove API is likely to be effective across a broader range of compounds. Given the use of shared lab equipment for multiple products, product-specific protocols are impractical. Thus, Oxcarbazepine serves as a conservative benchmark, a practice commonly endorsed in similar studies [21]. While other APIs may be evaluated in future work, establishing efficacy against the worst-case ensures robustness across all scenarios.

**Fig. 2:**
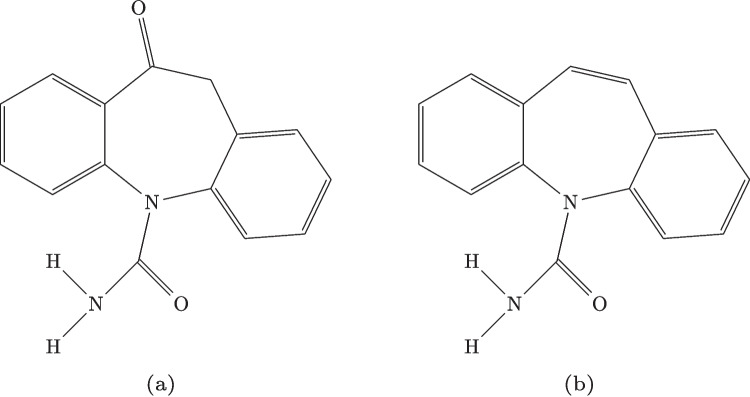
Chemical structures of: (**a**) Oxcarbazepine; (**b**) Carbamazepine

Oxcarbazepine (Oxc), with the chemical formula $\text {C}_{15}\text {H}_{12}\text {N}_{2}\text {O}_{2}$, is structurally illustrated in Fig. 2(a). It has a molecular weight of 252.27 g $\cdot$ mol$^{-1}$ and is a derivative of Carbamazepine, whose molecular structure is shown in Fig. 2(b). Oxc displays distinctive solubility properties. It is sparingly soluble in water, with a solubility of only 0.07 mg/mL at room temperature [22], classifying it as practically insoluble according to the scale proposed by Wolk et al. [23]. In contrast, it dissolves readily in certain organic solvents. For instance, at 35 $^{\circ }$C, its solubility in acetonitrile reaches 5.9 mg/mL, and in acetone, slightly higher at 6.5 mg/mL. When the temperature decreases to 15$^{\circ }$C, these values drop to 3.1 mg/mL and 3.6 mg/mL, respectively.

The concept of Residue Acceptable Limits (RALs) was introduced in the early 1990s [24]. In 1993, Fourman and Mullen [25] proposed specific contamination thresholds, including the widely referenced limit of no more than 10 ppm of a substance in another product. As cleaning validation protocols are primarily designed to prevent cross-contamination, evaluating residue levels involves determining the percent recovery from contact surfaces. Analytical methods must, therefore, offer sufficient sensitivity to detect residues at or below the defined RALs [26].

Although no official RAL exists for Oxcarbazepine in laboratory settings, it is essential to establish a practical and scientifically justified limit. Based on prior internal studies, the partnering pharmaceutical company has set the maximum allowable post-cleaning concentration for Oxc at 0.01 mg/mL (10 ppm). This value not only aligns with the guideline proposed by Fourman and Mullen [25] but also serves as a benchmark for validating the cleaning protocol’s effectiveness. Establishing this threshold ensures that equipment is adequately decontaminated and compliant with industry standards for residual control.

### Solvents for dissolving the API

The favorable solubility characteristics of the Oxc molecule in acetonitrile and acetone make these compounds as good candidates for incorporation into cleaning protocols.

Practically, acetonitrile and acetone were selected as analytical diluents for detecting residues of Oxc in equipment post-cleaning due to their high solubility for Oxc. Their use is well-established in cleaning validation protocols for production equipment. The selection of these solvents is intuitive for several reasons: (i) they are already established and routinely used in common laboratory activities; (ii) their inclusion does not necessitate additional contractual arrangements; (iii) they exhibit low toxicity compared to other organic solvents; and (iv) their cost falls within the budgetary constraints. Moreover, while there are other organic solvents with higher solubility for Oxc, they often fall short in meeting one or more of the aforementioned criteria.

Acetonitrile, chemically represented as CH$_{3}$CN, has a molecular mass of 41.05 g $\cdot$ mol$^{-1}$. At 25 $^{\circ }$C, its density is 0.781 g $\cdot$ cm$^{-3}$, and the vapor pressure at 20 $^\circ$C is 9.71 kPa. On the other hand, acetone, with the chemical formula C$_{3}$H$_{6}$O, has a molecular mass of 58.08 g $\cdot$ mol$^{-1}$, a density of 0.791 g $\cdot$ cm$^{-3}$ at 25 $^{\circ }$C, and a vapor pressure of 30.6 kPa at the same temperature. Acetonitrile and acetone used in this study were pro-analysis reagents. The acetonitrile was sourced from *PanReac Reagents*, with a purity larger than ${99.9}\,{\%\cdot (\text {v/v})}$, and the acetone from *Chem-Lab*, with a purity larger than ${99.5}\,{\%\cdot (\text {v/v})}$. The solvents were directly sampled from their respective flasks and used in the procedure.

These two compounds share similar physical properties, with acetone exhibiting slightly higher volatility. Moreover, acetone also demonstrates a slightly greater capacity for solubilizing Oxc compared to acetonitrile [22]. To summarize, acetonitrile and acetone are used as cleaning or rinsing agents to recover residual API from the equipment. The cleaning process, as described in Section “Application of the cleaning validation protocol”, incorporates a (different) detergent commonly used in current cleaning protocols.

### Sampling methods

The sampling of cleaned surfaces typically employs two primary techniques: swabbing and rinsing, as described by Food and Drug Administration et al. [4]. Swabbing is a direct surface sampling method, while rinsing is considered an indirect approach. The choice between these methods largely depends on the accessibility and geometry of the surface being evaluated. Swabbing is particularly effective for flat or irregular surfaces such as large panels and corners, whereas rinsing is more suitable for equipment with internal geometries such as pipes and tubes. In many laboratory-scale cleaning validations, a combination of both methods is often necessary to ensure comprehensive residue assessment.

#### Swab method

In this study, a polyester swab was selected for surface sampling due to its strength and consistency, following the guidance of Miscioscio [27]. This technique has been widely adopted and is detailed in several sources, including LeBlanc [8] and Bismuth and Neumann [12].

The swab is initially pre-wetted with an appropriate solvent, which plays a critical role in solubilizing surface-bound contaminants to enhance their transfer onto the swab. After pre-wetting, any excess solvent is removed, and the swab is systematically passed over a 100 cm$^2$ area using both horizontal and vertical strokes. To maximize collection efficiency, both sides of the swab are utilized, as suggested by Ovais et al. [28]. Following sampling, the swab is placed in a test tube containing solvent for a 10 min extraction period. The extract is then subjected to analytical procedures for residue quantification. Typical laboratory items assessed using the swab method include Petri dishes, spatulas, and mortars.

#### Rinse method

The rinse method involves washing contaminated equipment with a defined volume of solvent to ensure thorough contact with all surface areas. This procedure is performed at ambient temperature to support reproducibility. For consistency, each rinsing cycle is standardized to 10 s, and a total of 10 mL of solvent is used per equipment item.

The process begins by dispensing 5 mL of solvent onto the equipment surface, followed by agitation for 10 s. The resulting solution is collected as the primary rinse. This step is repeated with an additional 5 mL of solvent and agitation for the same duration. The solutions from both rinses are combined into a composite sample for analysis. This method is commonly used for equipment such as graduated cylinders, pycnometers, test tubes, and vials.

#### Swab absorption capacity

To determine the volume of solvent absorbed by a swab, a controlled test was performed. Initially, dry swabs were weighed to record their baseline mass, denoted as $W_d$. Each swab was then immersed in the solvent for approximately 3 s—sufficient time for full saturation—and weighed again to obtain the wet mass, $W_w$. The volume of solvent absorbed by the swab, $V$, was calculated using Eq. 1:

1 $$\begin{aligned} V = \frac{W_w - W_d}{\rho _s}, \end{aligned}$$

where $\rho _s$ is the solvent density (in g $\cdot$ mL$^{-1}$), measured at room temperature using the pycnometer method. This assessment was carried out using a set of 10 swabs for each solvent to ensure statistical robustness.

### Recovery study design

Recovery studies are a fundamental component of cleaning validation, as they demonstrate the ability to consistently recover residues from equipment surfaces [4]. The primary objective is to evaluate both the repeatability and efficiency of the sampling method—be it swabbing or rinsing—by quantifying the amount of analyte that can be reliably recovered from a known contaminated surface. These studies also help determine the optimal number of sampling steps needed to reach a satisfactory recovery rate.

Various regulatory guidelines provide differing thresholds for acceptable recovery. The Parenteral Drug Association (PDA) considers a recovery rate of 70 % to be excellent and 50 % to be acceptable [29]. According to the World Health Organization (WHO), values above 80 % are good, those between 50 % and 80 % are reasonable, and values below 50 % are questionable [30]. The Active Pharmaceutical Ingredients Committee (APIC) recommends a threshold of 90 % for exceptional recoveries and 50 % as the minimum acceptable level; values below this should be disregarded [31]. Forsyth [32] further suggests that operators aim for a recovery rate of at least 70 %, with a relative standard deviation not exceeding 15 %. Based on these recommendations, this study adopts a threshold of 70 % to indicate effective recovery.

#### Swab recovery test

The swab recovery test begins with the selection of an equipment surface—such as the bottom of a glass vessel or a stainless-steel plate—covering an area of approximately 100 cm$^2$. This surface is intentionally contaminated with a known volume of an Oxc solution of known concentration. After application, the solvent is allowed to evaporate completely in an oven. Once dried, the equipment is removed and cooled to ambient temperature.

A swab pre-moistened with the extraction solvent is then passed across the surface using a predefined pattern of horizontal and vertical strokes. The swab is subsequently placed into a vial containing solvent to dissolve the analyte. To maximize the recovery efficiency, a second swab is used on the same surface and processed under identical conditions. This secondary step aims to capture any remaining residues that were not collected in the first pass. Each vial is analyzed in duplicate to assess consistency.

#### Rinse recovery test

For the rinse recovery test, the contaminated glass or stainless-steel equipment is cleaned by sequential rinsing with two portions of the extraction solvent. Each rinsing step involves agitation for a fixed duration, and the collected liquids are pooled and transferred into test tubes for analysis.

#### Quantification and calculation of recovery rate

The amount of Active Pharmaceutical Ingredient (API) recovered via swabbing or rinsing is quantified using High Performance Liquid Chromatography (HPLC), as described later in Section “Chemical Analysis Techniques”. The recovery rate, $r_r$, expressed as a percentage, is calculated using Eq. 2:

2 $$\begin{aligned} r_r = \frac{m_{\text {Ex}}}{m_{\text {Co}}} \times 100~({\%}) \end{aligned}$$

where $m_{\text {Ex}}$ is the mass of Oxc recovered from the equipment surface (as determined by HPLC), and $m_{\text {Co}}$ is the mass originally applied during contamination.

#### Study scope and objective

Two different solvents were evaluated in this study to determine the most effective combination of solvent, recovery method, and equipment material. Although the final protocol is based on a single method-solvent pairing, the preliminary comparative assessment ensures that the choice is both data-driven and reproducible. The broader aim is to establish a validated procedure that can be easily adapted to other APIs and types of equipment with minimal modification.

In this context, validation of the cleaned equipment encompasses both the cleaning procedures and the sampling-recovery protocols. Therefore, in the remaining sections, this process is referred to as cleaning protocol validation.

#### Contaminant solution

The contaminant solution was prepared to match the maximum allowable concentration of Oxc on equipment surfaces, specifically 0.175 mg $\cdot$ mL$^{-1}$. The solvent used was the same mobile phase as employed in the HPLC analysis. Optimization of analytical component ranges was outside the scope of this study, as these parameters were established and validated in previous internal research. Our primary focus was the cleaning validation methodology, applying industry-standard practices that, while varying across companies, uniformly comply with international regulatory requirements. For brevity, chromatograms have been omitted from this report.

Solution stability regarding the API was ensured by: (i) performing analyses within the shortest feasible timeframe; (ii) inspecting solutions for any visible chemical degradation or phase separation prior to analysis; and (iii) storing solutions under appropriate conditions to prevent chemical or physical changes when immediate analysis was not possible. All solutions were stored at room temperature.

### Cleaning method in practice and validation strategy

This study validates the cleaning protocol depicted in Fig. 1, using Oxc as a worst-case contaminant. Validation was performed on both glassware and stainless-steel equipment. Two recovery methods—swabbing and rinsing—were evaluated to identify the most effective approach for contaminant removal. Recovery studies involved deliberate contamination of surfaces with Oxc solution, application of the cleaning protocol from Fig. 1, followed by assessment of recovery efficiency.

Two solvents, acetonitrile and acetone, were tested for their efficacy in dissolving and recovering the contaminant. Although only one solvent will be selected for routine use per equipment type, both were evaluated to ensure optimal choice. Similarly, one recovery method will ultimately be implemented for each equipment type after comparative assessment to confirm the most effective option.

### Testing the validation protocol in a realistic scenario

The cleaning validation methods described previously were applied to routine washing of laboratory equipment from both glassware and stainless steel categories, including items suited for swab and rinse methods.

Tests were conducted in duplicate (Replicate 1 and Replicate 2) alongside blanks (uncontaminated controls) to exclude accidental contamination. Each item was contaminated with 0.5 mL of the contaminant solution, dried at 60 $^{\circ }$C, cooled, then cleaned manually or automatically according to type. After a second drying cycle, sampling was performed using the recovery method and solvent chosen from prior recovery studies. Swabs were extracted in solvent vials and analyzed by HPLC.

To assess cleaning agent residues, rinses from two specimens per equipment type were analyzed by conductivity and Total Organic Carbon (TOC). For conductivity, rinsing used 5 mL purified water, and for TOC, 75 mL purified water was collected in nitric acid–washed flasks. The European Pharmacopoeia limits purified water conductivity to 4.3 µS $\cdot$ cm$^{-1}$ at 20 $^{\circ }$C and TOC to 500 ppm [33]. This study adopted a stricter cleaning agent residue limit of 10 ppm, per company standards.

### Chemical analysis techniques

This section summarizes the equipment and conditions used for the analysis of Oxc and cleaning agent residues.

Swab solvent volume was quantified gravimetrically using a *Mettler-Toledo ML204T/00* analytical balance.

HPLC analyses were performed on a *Waters Alliance e2695* system. The mobile phase consisted of 60 % eluent A (containing 0.14 % orthophosphoric acid and 0.10 % trimethylamine) and 40 % eluent B (acetonitrile), all volume/volume. Operating conditions were: (i) *Lichrospher 100 RP-8* column (250 mm $\times$ 4 mm $\times$ 5 µm), Merck; (ii) column and autosampler temperature at 25 $^{\circ }$C; (iii) detection wavelength: 215 nm; (iv) flow rate: 1.3 mL $\cdot$ min$^{-1}$; (v) injection volume: 20 µL; (vi) run time: 8 min; (vii) Oxc retention time: 3.5 min. The system was calibrated with standard solutions to correlate peak area at 215 nm to Oxc concentration. The validated method features a Limit of Quantification (LQ) of 0.005 µg $\cdot$ mL$^{-1}$, Limit of Detection (LD) of 0.002 µg $\cdot$ mL$^{-1}$, excellent linearity ($R^2=0.99997$), precision (RSD = 0.93 %), repeatability (RSD = 1.24 %), and a working range of 0.005 µg $\cdot$ mL$^{-1}$ 29.16 µg $\cdot$ mL$^{-1}$.

Conductivity was measured using a *Miron L® Ultrameter II 4P*, and Total Organic Carbon (TOC) was analyzed with an *Analytik Jena multi N/C 3100*.

### Statistical analysis

Descriptive statistics including minimum, maximum, mean ($\bar{x}$), standard deviation (SD), and coefficient of variation ($C_v$, %) were computed. $C_v$ quantifies repeatability and is defined as:

3 $$\begin{aligned} C_v = \frac{\text {SD}}{\bar{x}} \times 100~({\%}). \end{aligned}$$

A two-sample $t$-test was applied to assess whether differences in mean recovery rates—due to sampling method or solvent—are statistically significant at the 95 % confidence level [34, 35]. Specifically:

- $p < 0.05$: significant difference, favoring the alternative hypothesis;
- $p> 0.95$: supports the null hypothesis (no difference);
- $$0.05 \leqslant p \leqslant 0.95$$: inconclusive.

This approach provides clear insights into the comparative effectiveness of tested treatments.

## Results and discussion

### Swab absorption capacity

Table 8 (Appendix) reports the absorbed volume ($V$) from 10 samples. The absorption trends are consistent across both solvents, with minor fluctuations. Descriptive statistics in Table 1 show negligible differences in average absorbed volumes. The low standard deviation ($SD$) and coefficient of variation ($C_v$) confirm good repeatability.

A two-sample *t*-test indicates no significant difference in absorption between solvents, suggesting both are suitable for use. The final solvent choice should also consider factors such as toxicity, cost, and compatibility with specific equipment.

**Table 1:** Statistical summary of swab absorption volumes ($V$)

Solvent	$\mathbf {V_{\textrm{av}}}$ (mL)	$\mathbf {V_{\max }}$ (mL)	$\mathbf {V_{\min }}$ (mL)	$\textbf{SD}$ (mL)	$\mathbf {C_v}$ (%)
Acetonitrile	0.3378	0.3490	0.3230	0.0080	2.38
Acetone	0.3368	0.3489	0.3248	0.0091	2.70

$V_{\textrm{av}}$: average absorbed volume; $V_{\max }$, $V_{\min }$: maximum and minimum absorbed volumes; $SD$: standard deviation; $C_v$: coefficient of variation

Using the average absorption values, the maximum amount of contaminant dissolved by each solvent was estimated. Acetonitrile enables Oxc solubilization between 1.0 to 2.0 mg across temperatures 15 to 35 $^{\circ }$C, while acetone allows 1.2 to 2.1 mg. These limits guided the calibration range of the HPLC analysis.

### Recovery rate

The recovery study examined two solvents (acetonitrile and acetone), two types of equipment (glassware and stainless steel), and two sampling methods (swabbing and rinsing). Each cleaning procedure was performed twice—referred to as the 1st and 2nd cleanings throughout this paper—to assess the necessity of a second cleaning step. For each combination of solvent, equipment type, and sampling method, four replicates were conducted. All recovery rates are reported as percentages.

To improve readability, detailed results are presented in the Appendix (Table 9), while the discussion here focuses initially on the recovery rates obtained via the swab method. Table 2 summarizes key statistics of the recovery rates for the swab method. Both solvents achieved recovery rates above 70 % after the first cleaning, indicating that a second cleaning may be unnecessary. The first cleaning results also demonstrated good repeatability, with coefficients of variation ($C_v$) below 10 % for both solvents. In contrast, the second cleaning showed poor repeatability across all conditions.

**Table 2:** Statistical summary of recovery rates ($r_r$) obtained via the swab method

Solvent	Cleaning	$\mathbf {r_{r,\textrm{av}}}$ (%)	$\mathbf {r_{r,\max }}$ (%)	$\mathbf {r_{r,\min }}$ (%)	$\textbf{SD}$ (%)	$\mathbf {C_v}$ (%)
		Glassware Equipment				
Acetonitrile	1	80.38	83.69	76.71	3.63	4.52
Acetonitrile	2	8.32	10.93	5.63	3.01	36.21
Acetone	1	76.07	82.59	69.87	6.54	8.60
Acetone	2	27.07	27.85	26.67	0.67	2.49
		Stainless Steel Equipment				
Acetonitrile	1	90.00	92.92	87.11	3.10	3.44
Acetonitrile	2	4.63	6.69	2.60	2.34	50.63
Acetone	1	72.25	72.59	71.55	0.47	0.66
Acetone	2	11.21	11.31	11.06	0.12	1.06

$r_{r,\textrm{av}}$: average recovery rate; $r_{r,\max }$, $r_{r,\min }$: maximum and minimum recovery rates; $SD$: standard deviation; $C_v$: coefficient of variation

Acetonitrile shows superior performance for both types of equipment during the initial cleaning, while acetone also delivers acceptable recovery rates. The second cleaning compensates for the lower recovery rates observed after the first cleaning, resulting in nearly equivalent overall performance between the two solvents. Notably, acetone exhibits a higher recovery rate during the second cleaning, illustrating its effectiveness in this step.

Table 3 summarizes the statistical metrics for the recovery rates obtained via the rinse method. Several key observations emerge: (i) For glassware equipment, the recovery rates after the first cleaning remain consistently below 50 % regardless of the solvent used, indicating a clear advantage of the swab method over rinsing for this equipment type; (ii) For stainless steel equipment, recovery rates are generally below 75 % except when acetone is used, suggesting that acetone is the preferred solvent for rinsing stainless steel surfaces; (iii) When acetonitrile is used for rinsing stainless steel equipment, a second cleaning step appears necessary to improve recovery; (iv) The coefficient of variation for recovery rates after the first cleaning consistently remains below 10 %, reflecting good repeatability; and (v) Recovery rates from the second cleaning demonstrate reduced repeatability across the board.

**Table 3:** Statistical analysis of recovery rates ($r_r$) using the rinse method

Solvent	Cleaning	$r_{r,{\text {av}}}$ (%)	$r_{r,\max }$ (%)	$r_{r,\min }$ (%)	$SD$ (%)	$C_v$ (%)
		Glassware equipment				
Acetonitrile	1	46.19	49.73	42.71	4.02	8.69
Acetonitrile	2	23.84	26.93	20.78	3.53	14.82
Acetone	1	37.39	38.65	35.48	1.55	4.36
Acetone	2	26.32	35.48	17.15	10.58	61.67
		Stainless steel equipment				
Acetonitrile	1	65.09	67.92	62.93	2.34	3.59
Acetonitrile	2	9.39	11.15	8.34	1.33	14.16
Acetone	1	76.74	79.63	73.74	3.20	4.17
Acetone	2	37.36	38.10	36.70	0.63	1.68

$r_{r,{\text {av}}}$ - average recovery rate; $r_{r,\max }$ - maximum recovery rate; $r_{r,\min }$ - minimum recovery rate; $SD$ - standard deviation; $C_v$ - coefficient of variation

**Fig. 3:**
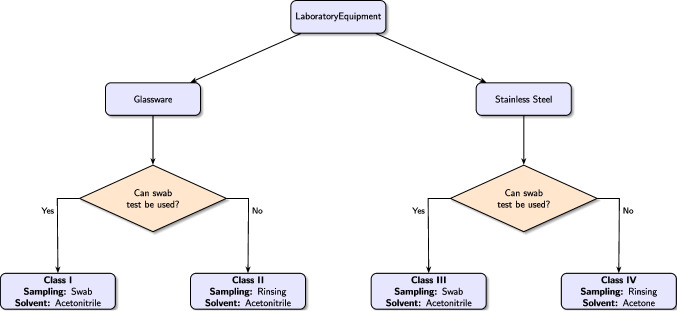
Flowchart depicting the decision process for selecting the appropriate cleaning procedure for each equipment type.

Current cleaning validation protocols generally recommend a minimum recovery rate of 50 %, with higher targets preferred for enhanced confidence. Our findings show that the cleaning methods and solvents tested consistently exceed these thresholds for dissolving the API. Specifically, as shown in Table 2, the swab method achieved recovery rates above 70 % for both solvents after the first cleaning. Meanwhile, Table 3 demonstrates that the rinse method attained recovery rates above 65 % when applied to stainless steel equipment. This comprehensive analysis informed the development of an optimized cleaning protocol, detailed in Fig. 3, allowing us to tailor the choice of solvents and recovery methods to each equipment type, thus meeting a primary objective of this study.

### Protocol development through statistical knowledge extraction

In this section, we utilize the previously presented data to perform hypothesis testing on the equality of mean recovery rates, applying the two-sample $t$-test as described earlier. Our goal is to statistically validate key observations identified previously by directly comparing the average recovery rates. This analysis focuses exclusively on the results from the first cleaning step.

We begin by testing the hypothesis that the mean recovery rate obtained via the swab method is greater than that achieved by the rinse method. Specifically, we investigate whether the swab method offers a statistically significant improvement over rinsing across different equipment types and solvents. For instance, consider the comparison between swab and rinse methods for glassware equipment using acetonitrile as the solvent. The resulting $p$-value is provided in the first column of Table 4, while the analogous value for acetone is shown in the second column. The findings demonstrate that, irrespective of the solvent, the swab method significantly outperforms rinsing for glassware equipment, strongly supporting the alternative hypothesis.

In the case of stainless steel equipment, the swab method shows a clear advantage when acetonitrile is used. Conversely, when acetone is the solvent, rinsing performs better, thus supporting the null hypothesis, as indicated by the $p$-value in the fourth column of Table 4.

**Table 4:** $p$-values from hypothesis tests comparing mean recovery rates during the first cleaning for swab versus rinse methods

Glassware Equipment		Stainless Steel Equipment	
Acetonitrile	Acetone	Acetonitrile	Acetone
$<0.0001$	$<0.0001$	$<0.0001$	0.0162$^{\ddagger }$

The hypotheses tested are $H_0:~r_{r,{\text {av}}}(\text {swab}) \leqslant r_{r,{\text {av}}}(\text {rinse})$ against $H_1:~r_{r,{\text {av}}}(\text {swab})> r_{r,{\text {av}}}(\text {rinse})$

$^{\ddagger }$ Indicates the alternative hypothesis $H_0:~r_{r,{\text {av}}}(\text {swab}) \geqslant r_{r,{\text {av}}}(\text {rinse})$ versus $H_1:~r_{r,{\text {av}}}(\text {swab}) < r_{r,{\text {av}}}(\text {rinse})$ is supported

Next, we assess whether acetonitrile offers an advantage over acetone for both types of equipment and sampling methods. The results, shown in Table 5, are interpreted similarly to those in Table 4. Key observations include: (i) acetonitrile is significantly more effective for glassware when using the rinse method, and for stainless steel equipment when employing the swab method; (ii) acetone demonstrates superior performance for stainless steel equipment when the rinse method is applied; and (iii) for glassware sampled with the swab method, both solvents exhibit comparable recovery rates.

**Table 5:** $p$-values from hypothesis tests comparing mean recovery rates during the first cleaning using acetonitrile versus acetone as solvents

Glassware Equipment		Stainless Steel Equipment	
Swabbing	Rinsing	Swabbing	Rinsing
0.1462	$<0.0001$	0.0032	0.0005$^{\ddagger }$

The hypotheses tested are $H_0:~r_{r,{\text {av}}}(\text {acetonitrile}) \leqslant r_{r,{\text {av}}}(\text {acetone})$ versus $H_1:~r_{r,{\text {av}}}(\text {acetonitrile})> r_{r,{\text {av}}}(\text {acetone})$

$^{\ddagger }$ Indicates support for the alternative hypothesis $H_0:~r_{r,{\text {av}}}(\text {acetonitrile}) \geqslant r_{r,{\text {av}}}(\text {acetone})$ versus $H_1:~r_{r,{\text {av}}}(\text {acetonitrile}) < r_{r,{\text {av}}}(\text {acetone})$

Based on these results, laboratory equipment was classified into four categories considering: (i) the type of equipment: glassware or stainless steel; and (ii) its suitability for sampling using the swab method. The resulting classification is: (i) Class I — glassware equipment suitable for swab sampling; (ii) Class II — glassware equipment unsuitable for swab sampling; (iii) Class III — stainless steel equipment suitable for swab sampling; and (iv) Class IV — stainless steel equipment unsuitable for swab sampling.

Building on this classification, we propose the following guidelines for implementing the cleaning efficiency assessment protocol for common laboratory equipment:

1. For equipment in Classes I and III, apply the swab method using acetonitrile as the solvent;
2. For equipment in Class II, use the rinse method with acetonitrile as the solvent;
3. For equipment in Class IV, employ the rinse method with acetone as the designated solvent.

These guidelines are summarized schematically in Fig. 3 and are evaluated in the subsequent section.

## Application of the cleaning validation protocol

This section presents an overview of the results obtained by applying the previously outlined guidelines to validate the cleaning process of laboratory equipment, encompassing both glassware and stainless steel categories.

### Detection of API residues on equipment surfaces

A total of 17 pieces of laboratory equipment were randomly selected for protocol validation under realistic conditions. These items represent the laboratory’s most frequently used equipment, providing a diverse sample to enhance the generalizability of our findings. Each piece was initially contaminated with a solution of Oxcarbazepine (Oxc) and subsequently cleaned following established manual and automated washing procedures. Post-cleaning, residue sampling was performed using both swab and rinse methods with the appropriate solvents. The collected samples were analyzed via HPLC to quantify residual Oxc concentrations. To ensure robust repeatability, two replicates of each equipment type were tested, with each collected solution measured twice. The percent relative standard deviation ($\%\textrm{RSD}$) of the Oxc signal did not exceed 2 %.

It is important to note that API evaporation during drying can compromise residue recovery accuracy, particularly for glassware surfaces. However, this effect was not observed in our study due to Oxc’s high vaporization temperature (approximately 200 $^{\circ }$C), which prevents significant evaporation under typical drying conditions. According to the protocol developed through statistical analysis, future implementation requires testing each equipment item with only one sampling method and solvent. Nevertheless, during this validation phase, both sampling methods and solvents were employed for all items.

Table 6 summarizes the results. The second column classifies equipment into categories (I, II, III, IV), which determines the sampling method and solvent used. For the majority of items, Oxc was undetectable after cleaning. The only exception was test tubes, where trace amounts of Oxc were detected in certain replicates; however, concentrations remained well below the established acceptance limit of 0.01 mg $\cdot$ mL$^{-1}$.

**Table 6:** API contamination results following equipment cleaning (Oxcarbazepine concentrations in mg $\cdot$ mL$^{-1}$)

		Solvent: Acetonitrile				Solvent: Acetone			
		Replicate 1		Replicate 2		Replicate 1		Replicate 2	
Equipment Item	Class	Meas. 1	Meas. 2	Meas. 1	Meas. 2	Meas. 1	Meas. 2	Meas. 1	Meas. 2
Beaker (25 mL)	I	Undet.	Undet.	Undet.	Undet.	Undet.	Undet.	Undet.	Undet.
Beaker (250 mL)	I	Undet.	Undet.	Undet.	Undet.	Undet.	Undet.	Undet.	Undet.
Dissolution vessel (1 L)	I	Undet.	Undet.	Undet.	Undet.	Undet.	Undet.	Undet.	Undet.
Dissolution basket	III	Undet.	Undet.	Undet.	Undet.	Undet.	Undet.	Undet.	Undet.
Dissolution paddle	IV	Undet.	Undet.	Undet.	Undet.	Undet.	Undet.	Undet.	Undet.
Glass mortar (100 mm)	I	Undet.	Undet.	Undet.	Undet.	Undet.	Undet.	Undet.	Undet.
Petri plate (60$\times$15 mm)	I	Undet.	Undet.	Undet.	Undet.	Undet.	Undet.	Undet.	Undet.
Petri plate (100$\times$20 mm)	I	Undet.	Undet.	Undet.	Undet.	Undet.	Undet.	Undet.	Undet.
Volumetric pipette (1 mL)	II	Undet.	Undet.	Undet.	Undet.	Undet.	Undet.	Undet.	Undet.
Volumetric pipette (25 mL)	II	Undet.	Undet.	Undet.	Undet.	Undet.	Undet.	Undet.	Undet.
Volumetric balloon (10 mL)	II	Undet.	Undet.	Undet.	Undet.	Undet.	Undet.	Undet.	Undet.
Volumetric balloon (100 mL)	II	Undet.	Undet.	Undet.	Undet.	Undet.	Undet.	Undet.	Undet.
Pycnometer	II	Undet.	Undet.	Undet.	Undet.	Undet.	Undet.	Undet.	Undet.
Graduated cylinder (10 mL)	II	Undet.	Undet.	Undet.	Undet.	Undet.	Undet.	Undet.	Undet.
Graduated cylinder (100 mL)	II	Undet.	Undet.	Undet.	Undet.	Undet.	Undet.	Undet.	Undet.
Test tube (18$\times$180 mm)	II	Undet.	Undet.	0.00134	0.00109	0.00032	Undet.	0.00133	0.00144
Test tube (25$\times$150 mm)	II	Undet.	Undet.	Undet.	Undet.	0.00639	0.00248	Undet.	Undet.

*Undet.* denotes “Undetected”

### Detection of detergent residues on equipment surfaces

Despite effective removal of the active pharmaceutical ingredient (API), detergent residues—especially surfactants—may persist on equipment surfaces after cleaning. To assess the efficiency of detergent removal, rinsing solutions were analyzed for conductivity and Total Organic Carbon (TOC), with results summarized in Table 7. Conductivity values (columns three and four) are presented as averages with 95 % confidence intervals (±), including the analysis temperature. TOC concentrations appear in the last two columns.

All conductivity measurements fall well below the purified water limit of 4.3 µS $\cdot$ cm$^{-1}$ at 20 $^{\circ }$C, indicating negligible ionic contamination. Most TOC values are below the analyzer’s quantification limit (0.500 ppm); those above remain significantly under the 10 ppm acceptance threshold. These findings confirm the cleaning procedure’s effectiveness in removing detergent residues.

**Table 7:** Conductivity and Total Organic Carbon (TOC) analysis of rinsing water samples

Equipment Item	Class	Conductivity (µS $\cdot$ cm$^{-1}$)		TOC (ppm)	
		Replicate 1	Replicate 2	Replicate 1	Replicate 2
Beaker (25 mL)	I	1.17(0.04) (21.5 $^{\circ }$C)	1.11(0.04) (21.5 $^{\circ }$C)	$<\text {LQ}$	$<\text {LQ}$
Beaker (250 mL)	I	0.73(0.01) (20.9 $^{\circ }$C)	0.69(0.01) (20.9 $^{\circ }$C)	$<\text {LQ}$	$<\text {LQ}$
Dissolution vessel (1 L)	I	1.25(0.07) (20.5 $^{\circ }$C)	1.06(0.01) (20.2 $^{\circ }$C)	$<\text {LQ}$	$<\text {LQ}$
Dissolution basket	III	1.05(0.04) (20.0 $^{\circ }$C)	1.06(0.01) (20.2 $^{\circ }$C)	$<\text {LQ}$	$<\text {LQ}$
Dissolution paddle	IV	0.96(0.03) (20.0 $^{\circ }$C)	0.80(0.03) (19.9 $^{\circ }$C)	$<\text {LQ}$	$<\text {LQ}$
Glass mortar (100 mm)	I	1.77(0.03) (20.7 $^{\circ }$C)	1.30(0.02) (20.8 $^{\circ }$C)	$<\text {LQ}$	$<\text {LQ}$
Petri plate (60$\times$15 mm)	I	0.75(0.04) (20.6 $^{\circ }$C)	0.78(0.06) (21.0 $^{\circ }$C)	0.510	$<\text {LQ}$
Petri plate (100$\times$20 mm)	I	1.97(0.04) (21.4 $^{\circ }$C)	0.79(0.05) (21.0 $^{\circ }$C)	0.671	$<\text {LQ}$
Volum. pipette (1 mL)	II	0.99(0.01) (20.2 $^{\circ }$C)	0.96(0.03) (20.3 $^{\circ }$C)	$<\text {LQ}$	0.624
Volum. pipette (25 mL)	II	1.05(0.01) (20.3 $^{\circ }$C)	1.15(0.01) (20.1 $^{\circ }$C)	3.590	1.090
Volum. balloon (10 mL)	II	0.85(0.01) (20.0 $^{\circ }$C)	0.92(0.01) (20.1 $^{\circ }$C)	$<\text {LQ}$	$<\text {LQ}$
Volum. balloon (100 mL)	II	0.93(0.03) (20.4 $^{\circ }$C)	0.95(0.03) (20.1 $^{\circ }$C)	$<\text {LQ}$	$<\text {LQ}$
Pycnometer	II	2.17(0.01) (20.6 $^{\circ }$C)	1.04(0.02) (20.5 $^{\circ }$C)	$<\text {LQ}$	$<\text {LQ}$
Grad. cylinder (10 mL)	II	0.85(0.01) (20.5 $^{\circ }$C)	0.92(0.01) (20.1 $^{\circ }$C)	$<\text {LQ}$	$<\text {LQ}$
Grad. cylinder (100 mL)	II	0.93(0.03) (20.4 $^{\circ }$C)	0.95(0.03) (20.4 $^{\circ }$C)	$<\text {LQ}$	$<\text {LQ}$
Test tube (18$\times$180 mm)	II	0.78(0.04) (21.0 $^{\circ }$C)	0.74(0.05) (21.0 $^{\circ }$C)	$<\text {LQ}$	$<\text {LQ}$
Test tube (25$\times$150 mm)	II	0.80(0.01) (21.5 $^{\circ }$C)	0.82(0.03) (21.9 $^{\circ }$C)	$<\text {LQ}$	$<\text {LQ}$

$<\text {LQ}$ indicates a measure below the quantification limit of the TOC analyzer

Summarizing, the conductivity measurements of the rinsing solutions and Total Organic Carbon analyses collectively confirm the cleaning procedure’s effectiveness, thereby validating its overall efficacy.

### Limitations and future work

This study successfully validates a cleaning protocol using Oxcarbazepine as a worst-case model compound; however, several limitations should be noted. Validation was performed with a single API and a limited variety of equipment surfaces under controlled laboratory conditions. Despite this, we do not anticipate major restrictions when applying the protocol more broadly, aside from the potentially significant labor involved. The choice of Oxcarbazepine—a poorly soluble and challenging-to-clean compound—provides a robust scientific basis for extending the protocol to less demanding substances.

Future work should aim to verify the protocol’s robustness across a wider spectrum of APIs, particularly those differing in physicochemical properties, potency, and toxicological profiles. Additionally, evaluation on a broader range of surface materials, equipment geometries, and hard-to-clean or inaccessible areas would be valuable. Investigating the impact of variable cleaning conditions will further assess the protocol’s resilience under operational variability.

Moreover, developing adaptive, risk-based cleaning strategies tailored to specific scenarios is recommended. Increasing the number of repetitions, expanding equipment sampling, and including batch-to-batch variability will improve statistical reliability. Finally, while the current analytical methods effectively detected Oxcarbazepine and surfactant residues, future studies could benefit from more sensitive or multi-residue analytical techniques (e.g., LC-MS/MS) to detect a wider array of contaminants, including degradation products and excipients [36].

## Conclusions

Cleaning validation is a critical component of a robust compliance program in regulated pharmaceutical environments. It provides documented assurance that approved cleaning procedures consistently remove residues of prior products or cleaning agents from equipment, maintaining levels below scientifically established maximum allowable carryover limits. Widely recognized by both regulatory authorities and industry, cleaning validation plays a vital role in controlling product cross-contamination.

In recent years, the scope of cleaning validation has expanded to include activities within quality control laboratories. However, comprehensive case studies detailing the systematic development of cleaning validation protocols specifically for laboratory equipment remain scarce. This gap is further amplified by the need to adapt protocols to existing industry constraints and procedures.

This study presents a structured methodology to address these challenges, comprising the following key steps: (i) identification of a worst-case API to guide protocol development; (ii) determination of the optimal sampling solvent and method for each equipment class via recovery studies; and (iii) use of the gathered data and analytical tools to design a tailored validation protocol.

The proposed approach was tested in a real-world pharmaceutical setting, with Oxcarbazepine identified as the worst-case API. Recovery studies informed the selection of appropriate sampling methods and solvents. The cleaning validation was conducted along two main lines: (i) analysis of API residues on equipment surfaces after routine cleaning; and (ii) detection of detergent residues through conductivity and Total Organic Carbon (TOC) measurements.

Both assessments conclusively demonstrated the cleaning procedure’s effectiveness, validating the protocol. We believe this scientifically grounded approach offers broad applicability across diverse scenarios and organizations, though successful implementation requires adaptation to each company’s specific context and culture.

## Data Availability

The data supporting the findings of this study are available within the paper.

## Appendix

### A.1: Comparison of swab absorption for different solvents

**Table 8:** Swab absorption capacities using acetonitrile and acetone (in mL)

	Sample									
**Solvent**	1	2	3	4	5	6	7	8	9	10
Acetonitrile	0.3412	0.3490	0.3436	0.3424	0.3311	0.3230	0.3287	0.3441	0.3363	0.3386
Acetone	0.3369	0.3280	0.3335	0.3489	0.3478	0.3267	0.3404	0.3475	0.3248	0.3331

### A.2: Comparison of recovery methods

**Table 9:** Recovery rates comparison between swab and rinse sampling methods (in %)

		Glassware equipment		Stainless steel equipment	
Solvent	Sample	1st cleaning	2nd cleaning	1st cleaning	2nd cleaning
		Sampling method: swabbing			
Acetonitrile	1	77.81	10.92	87.11	6.63
Acetonitrile	2	76.71	10.93	87.54	6.69
Acetonitrile	3	83.69	5.63	92.92	2.60
Acetonitrile	4	83.31	5.79	92.43	2.60
Acetone	1	69.87	27.85	71.55	11.06
Acetone	2	71.04	26.70	72.46	11.17
Acetone	3	80.76	26.67	72.59	11.31
Acetone	4	82.59	47.40	72.41	11.30
		Sampling method: rinsing			
Acetonitrile	1	49.60	20.78	67.92	8.34
Acetonitrile	2	49.73	20.78	66.07	8.38
Acetonitrile	3	42.71	26.93	62.93	9.67
Acetonitrile	4	42.71	26.87	63.44	11.15
Acetone	1	36.79	35.48	73.74	37.62
Acetone	2	35.48	35.48	74.20	38.10
Acetone	3	38.65	17.17	79.37	37.00
Acetone	4	38.65	17.15	79.63	36.70
